# Municipal Risk Communication and Public Trust: Reducing Counterproductive Behaviors Across Emergency Scenarios

**DOI:** 10.1111/risa.70232

**Published:** 2026-03-30

**Authors:** Moran Bodas, Omar Watad, Stav Shapira

**Affiliations:** ^1^ Department of Emergency and Disaster Management School of Public Health Gray Faculty of Medical and Health Sciences Tel Aviv University Tel Aviv‐Yafo Israel; ^2^ School of Public Health Faculty of Health Sciences Ben‐Gurion University of the Negev Be'er‐Sheva Israel

**Keywords:** Behavior, municipal authorities, public trust, risk communication, rumor control

## Abstract

Municipal authorities play a critical role in risk communication, yet little is known about how their messages influence public safety behavior. This study examined how municipal informational messages shaped behavioral intentions across five emergency scenarios (wildfires, flooding, hazardous materials, extreme weather, and civil unrest) in a representative sample of 1509 adults. Using a cross‐sectional experiment, we compared participants exposed to rumors alone versus rumors with municipal guidance. Results show that municipal messaging significantly reduced counterproductive behaviors such as rumor‐spreading and convergence on hazard sites, though it had limited effects on preparedness and proactive information seeking. Trust in national authorities remained stronger than in local governments, and demographic factors such as gender, age, and political orientation moderated behavioral responses. These findings extend established models of risk communication by highlighting the contextual role of municipal authorities and suggest that local communication strategies are best framed as harm‐reduction tools. The study underscores the need for scenario‐specific message design, proactive trust‐building, and future field‐based research to capture real‐world behaviors.

## 1 Introduction

Emergencies and disasters exact a substantial toll in lives and property annually (Kelman 2020). Unprepared populations are disproportionately affected and face greater recovery challenges (Marshall et al. 2020; Ide 2023). Thus, resilience and preparedness—grounded in access to reliable, lifesaving information—are vital to enhance public coping during crises (Ntontis et al. 2021). In emergencies, the public's demand for information surges due to heightened uncertainty and the need to comprehend threats and appropriate responses (Rubin et al. 2012). Effective communication must offer clear “do” and “don't” guidance to support safety and decision‐making (Fakhruddin et al. 2020).

Research consistently shows that clear, consistent guidance fosters more effective public response, strengthening resilience (Andreassen et al. 2020; Kankanamge et al. 2020). In contrast, contradictory or vague messages erode public trust and reduce coping ability. These reactions are tied to the science of threat perception, which explores how individuals process fear, often driven more by emotion than rational analysis (Gray and Ropeik 2002). Key factors influencing perception include the threat's perceived severity, likelihood, intrusiveness, controllability, and familiarity (Gray and Ropeik 2002; Bodas et al. 2015). Public appraisal of response actions also matters. Perceived appropriateness, self‐efficacy, and cost‐benefit considerations all shape reactions to official guidance (Paek et al. 2010).

In crises, individuals engage two cognitive mechanisms: the intuitive “feelings as risk” and the deliberative “analysis as risk” (Gray and Ropeik 2002; Acton et al. 2007; Rogers et al. 2007). Although emotional reactions often precede analytical ones, prior knowledge, a sense of control, and effective communication can shift people toward rational decision‐making. Understanding these motivations and behavioral drivers is central to optimizing emergency risk communication (Rogers et al. 2007; Sheppard 2011).

### 1.1 Risk Communication in Emergencies

Risk communication—a cornerstone of emergency management—refers to the process of delivering information to the public under uncertain and threatening conditions. Its success hinges on clarity, speed, and accuracy (Gray and Ropeik 2002). Effective communication directly influences public understanding and behavior during emergencies (Gray and Ropeik 2002; Rogers et al. 2007; Sheppard 2011; Rogers et al. 2013), while ineffective systems can worsen crises and strain response operations (Gray and Ropeik 2002; Acton et al. 2007).

Accurate messaging also helps maintain order and trust, preventing rifts between the public and institutions (Gray and Ropeik 2002). For example, during the 2022 Russian attacks on Odesa, poor guidance and inconsistent messaging caused widespread scare, overcrowding in makeshift shelters, and increased public health risks (Sellnow and Seeger 2021; Zhang and Zhou 2023). Similarly, during COVID‐19, inconsistent global communication led to misinformation, conspiracy theories, and widespread non‐compliance with safety measures like vaccination and social distancing (Tuğlular et al. 2023). Research identifies message timeliness, consistency, and rumor control as key factors in communication success (Latkin et al. 2023).

Sociodemographic variables, especially age and socioeconomic status, influence public reactions. Older adults may have less access to certain information sources and be more influenced by the “digital divide,” while younger populations tend to access more information sources and remain more engaged (Ghio et al. 2021; Tyler and Sadiq 2018; Chu et al. 2021; Van Deursen 2020). Gender also plays a role. In many cases, individuals responsible for caregiving roles—often women—demonstrate heightened concern for personal and family safety and are more likely to adhere to protective behaviors. Men, by contrast, may exhibit skepticism and overconfidence, making them more susceptible to misinformation and slower to follow guidance (Chen and Cong 2024; Gündüz 2024; Paimre and Osula 2022). These variations affect how populations respond to wartime messaging and underline the need for tailored communication strategies.

### 1.2 Social Media and Its Impact on Risk Communication

The rise of social media platforms such as Facebook, X (formerly Twitter), WhatsApp, and Telegram has transformed emergency communication (Link and Baumann 2023). These platforms allow real‐time dissemination of critical information across wide audiences (Guzek et al. 2020; Hagar 2013; Roy et al. 2020), improving response efficiency during crises like pandemics, wars, and natural disasters (Ghio et al. 2021; Kankanamge et al. 2020; Hao and Wang 2020; Saroj and Pal 2020; Karami et al. 2020; Yeshua‐Katz et al. 2025). For instance, during the 2013 Nairobi terrorist attack, the Kenyan police used X (then Twitter) extensively, issuing over 560 tweets, far outnumbering Facebook posts (Simon et al. 2014).

However, social media's openness also facilitates the spread of rumors, disinformation, and fake news (Fard and Verma 2022; Kaur and Gupta 2023). Following the 2013 Boston Marathon bombing, Twitter was flooded with false reports, including fabricated details about victims and perpetrators (Starbird et al. 2014). Similar issues plagued responses to Hurricanes Harvey and Irma in 2017 (Hunt et al. 2020), as well as the COVID‐19 pandemic, where a flood of mixed messages hindered public understanding (Wong et al. 2021). Repeated exposure to online misinformation can lead users to perceive it as credible and share it further, fueling its viral spread (Pundir et al. 2021). Research differentiates platforms in terms of their misinformation potential: while Facebook saw a decline in fake news post‐2017, X did not (Allcott et al. 2019). WhatsApp now experiences the most rapid growth in misinformation (Tandoc 2020).

Despite this, research on information‐seeking patterns shows a complex picture. While many people continue to seek reliable information sources (Bodas et al. 2015; Simon et al. 2014), the COVID‐19 pandemic appears to have shifted these patterns, particularly among younger demographics. Recent research indicates increased use of and trust in social media as an information source, especially among younger adults, alongside documented declines in trust in experts and authorities in some contexts (Pew Research Center 2025). However, these trends vary significantly across populations and contexts. Studies conducted prior to the pandemic found that only a small minority (approximately 6%) relied solely on social media (Simon and Goldberg 2015; Simon et al. 2015), though this figure likely warrants reassessment in light of recent changes. Messages from credentialed experts and official authorities remain influential for many populations (Liu et al. 2016; Simon et al. 2014), though their perceived trustworthiness may be more contested than in previous decades. Emergency services can still leverage institutional credibility to counter misinformation, but must do so with awareness of decreased baseline trust and increased skepticism, particularly among populations that experienced institutional failures or inconsistent messaging during the pandemic (Simon et al. 2015; Simon et al. 2014; Hakak et al. 2020). This evolving trust landscape underscores the importance of municipal authorities establishing and maintaining credibility during non‐emergency periods.

### 1.3 The Role of Local Authorities in Risk Communication

For the purposes of this study, we define “local authorities” as municipal and regional government bodies responsible for service delivery and emergency management at the community level. In the Israeli context, this includes municipal governments (municipalities and regional councils) and their associated departments, such as municipal emergency management units, local spokesperson offices, and community services. This excludes national agencies such as the Israel Police, Magen David Adom (national emergency medical services [EMS]), and the Israel Fire and Rescue Services, which operate under national command structures despite having local stations. This distinction is important because Israel operates under a unitary government system, where local authorities have more limited autonomy and resources compared to federal systems, yet are still expected to serve as primary communicators during localized emergencies. When we refer to “national authorities” or “national emergency bodies,” we mean centrally governed organizations such as the Home Front Command (Israel's civil defense body), the Israel National Police, national EMS, and national fire and rescue services.

Trust in authorities during emergencies is not monolithic; rather, it varies by level of government and type of emergency. Research has documented that public trust in local authorities often differs from trust in national or federal agencies, though the direction and magnitude of this difference depend on context, governance structure, and prior experience. In some settings, local authorities benefit from proximity and perceived responsiveness, generating higher trust than distant national agencies (Wachinger et al. 2013). In others, national authorities may be perceived as more resourced, professional, or politically neutral (Siegrist and Cvetkovich 2000). During the COVID‐19 pandemic, trust patterns varied considerably: some communities reported higher confidence in local public health officials who provided tailored guidance, while others looked to national bodies for authoritative scientific information (Plohl and Musil 2021). Understanding these trust dynamics is critical, as messages from distrusted sources may be ignored or actively resisted, while trusted messengers can promote compliance even with difficult or inconvenient directives. Given this context, examining how local authorities’ communications influence public behavior—and how that influence compares to national‐level messaging—represents an important gap in the literature.

Local authorities are uniquely positioned to deliver risk communication effectively. As the governmental body most directly engaged with the public, they are essential for relaying critical preparedness and response messages Balog‐Way et al. 2020; MacKay et al. 2022). Their proximity enables targeted, accessible communication tailored to community needs.

Local authorities often initiate early warnings, coordinate evacuations, and manage local resources. Their effectiveness hinges on timely and clear communication (Sufri et al. 2020). For instance, in the United States, clear local guidance during hurricanes improves safety compliance and reduces casualties (Collins et al. 2021). In Japan, local governments play a key role in tsunami preparedness using tools like alarms and social media (Mavrodieva and Shaw 2021).

Their responsibilities also include coordination with national agencies and managing health campaigns. During the COVID‐19 pandemic, local authorities led testing, vaccination, and enforcement efforts. In South Korea, mobile alerts and frequent updates enhanced compliance and curbed infection rates (Im and Campbell 2020). In New Zealand, close collaboration with national agencies ensured successful lockdown communication (Anttiroiko 2021). Their role extends beyond immediate emergency response to include the dissemination of accurate information, the prevention of misinformation, and the provision of psychosocial support. For example, after the 2015 Paris attacks, local authorities collaborated with national bodies to provide real‐time information and safety guidelines, helping to reduce public anxiety and confusion. Studies emphasize that clear, authoritative communication is essential for alleviating public distress and enhancing community resilience (Garcia and Rimé 2019).

Communication strategies now include a wide range of channels—traditional media, social media, and direct outreach through community leaders. Research shows that social platforms like X and Facebook enhance awareness and speed of information during crises (Hyland‐Wood et al. 2021; Lovari and Valentini 2020). To ensure inclusivity, local authorities also work with community organizations to reach vulnerable or marginalized populations, such as non‐native speakers.

In the Israeli context specifically, the public's relationship with emergency communication has been shaped by decades of experience with recurring security threats. Qualitative research has documented what Shapiro and Bird‐David (2017) term “routinergency”—a state in which emergency preparedness becomes routinized into everyday life, creating unique patterns of public response. This routinization can produce both adaptive behaviors (such as rapid compliance with familiar protocols) and maladaptive ones (such as complacency or selective attention). Understanding these dynamics is particularly important for municipal authorities, who must craft messages that penetrate this state of normalized alert while avoiding both panic and dismissiveness. This context makes Israel a particularly relevant case study for examining how local authorities communicate risk in environments where emergencies are not exceptional events but recurring features of civic life.

Building on this literature, the present study aimed to examine how municipal informational messages influence public behavioral intentions during emergencies. Specifically, we investigated the following research questions: RQ1: How does exposure to local authority guidance reduce counterproductive behaviors (such as rumor‐spreading and convergence on hazard sites) compared to exposure to rumors alone?; RQ2: How does exposure to local authority guidance enhance preparedness and knowledge‐seeking actions?; RQ3: How do trust in authorities (local vs. national) and sociodemographic factors (such as gender, age, political orientation) shape behavioral responses to emergency communications?; and RQ4: How do behavioral responses vary systematically across different types of emergencies (natural‐technological events vs. human‐induced events)?

## 2 Methods

### 2.1 Study Design

The study conducted is a cross‐sectional interventional study based on comparison groups. As the study involved reading text passages, an online format was chosen to facilitate participant recruitment, allocation to groups, and implementation of the experimental and control conditions.

### 2.2 Population and Sampling

The study population in the survey comprises the entire adult Israeli public (age 18 and above). Inclusion criteria include: adults (aged 18 and above), Hebrew speakers, and members of an online panel that enables rapid and high‐quality sampling.

**FIGURE 1:**
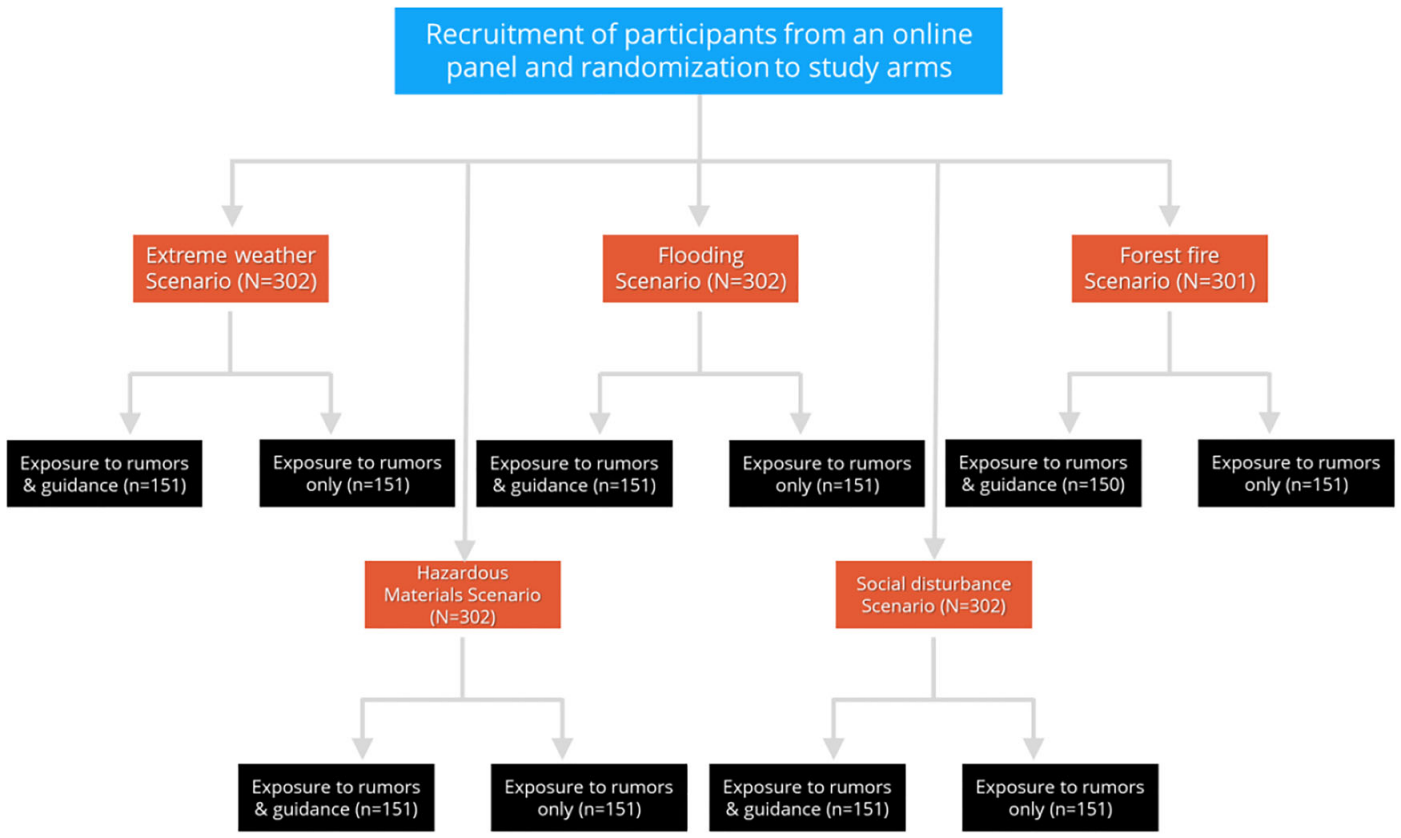
Flowchart of study arms.

Participant recruitment was carried out from a pool of panelists enlisted on the panel service of a polling service (“Sekernet”), which includes 50,000 Israelis from across the spectrum of Israeli society. Recruitment was conducted according to predefined rules and quotas to ensure appropriate representation based on gender, age, place of residence, education, and income. After recruitment, participants were randomized into the study arms (Figure 1).

In a previous study conducted by the research team (Bodas et al. 2021), it was found that the average level of counterproductive behavioral intention in the control group was 3.48 (SD = 1.06), compared to an average of 2.92 (SD = 1.27) in the group exposed simultaneously to rumors, informational campaigns, and official guidelines. According to the OpenEpi calculator, the minimum required sample size for detecting such a mean difference in a population exceeding 10 million people, with a significance level of 95% and statistical power of 80%, is 138. Accordingly, each arm of the study (see Figure 1) included at least 150 participants, with a total of 1500 participants (the final sample included 1509 participants).

### 2.3 Variables and Tools

#### 2.3.1 Independent Variables

The primary independent variable in this study is the type of intervention (exposure to rumors vs. exposure to rumors accompanied by guidance and explanation). A secondary independent variable is the type of emergency scenario (see Figure 1). The intervention text for scenario presentation, rumors, and guidance and explanations is provided in Appendix 1 of the Supporting Information.

#### 2.3.2 Dependent Variable

The primary dependent variable is **behavioral intention**. Behavioral intention includes several items formulated specifically for the present study based on the findings of an expert panel. These items examine the respondent's intention to perform various behaviors—some desirable and some counterproductive—using a Likert scale ranging from 1 (not at all) to 5 (to a very large extent).

In addition, potential **mediating variables** in the relationship between the independent and dependent variables were measured, including:

- **Perceived Threat (7 Items)**: These measures assess perceived likelihood, severity, and threat intrusiveness regarding emergency situations, as well as trust in the guidelines and response forces. The measures are based on previous studies in the field (Bodas et al. 2015). The two items related to perceived severity demonstrated high reliability in the present study (Cronbach's alpha = 0.821).
- **Trust Measures**: Trust was measured through multiple items assessing different dimensions. First, trust in emergency guidelines was assessed through the item: “To what extent do you trust the emergency guidelines provided in the scenario?” (measured on a 5‐point Likert scale from 1 = Not at all to 5 = Very much). Second, trust in first responders' capacity to respond was assessed through: “To what extent do you trust the ability of emergency and rescue services to respond effectively to this type of emergency?” (measured on the same 5‐point scale). Third, we assessed participants’ general trust in local authorities versus national authorities through two items: “I would seek guidance from my local municipality/local authority” and “I would contact national emergency organizations (such as police, fire services, or Magen David Adom)” (both measured as behavioral intentions on the same 5‐point scale). In addition, we included a contextual measure: trust in government during the “Iron Swords” war (the ongoing conflict at the time of data collection), assessed through a single item on a 5‐point scale. These measures allowed us to distinguish between trust in message content, trust in institutional capacity, and trust in specific governmental levels—all of which have been shown to independently influence compliance behaviors (Siegrist and Cvetkovich 2000; Wachinger et al. 2013).
- **Classification of Authorities**: To avoid confusion, we explicitly define how various emergency response entities were classified in this study. Local authorities were defined as municipal government and spokesperson, local emergency management units, regional councils, and municipal services (water, sanitation, etc.), whereas National authorities were defined as the Israel Police, Israel Fire and Rescue Services, Magen David Adom (national EMS), Home Front Command (the civil defense body), and national government ministries and agencies. When the text refers to “emergency and rescue services” or “first responders,” these terms encompass national‐level entities (police, fire, EMS) operating under centralized command, distinct from municipal authorities. This distinction reflects the Israeli unitary government structure, where emergency response services operate as national agencies despite having local stations.
- **Sense of Preparedness (3 Items)**: The sense of preparedness index is based on the participant's average response to three statements assessing knowledge, psychological preparedness, and physical preparedness. These are measured on a Likert scale from 1 (Not at all) to 5 (Very much). In the current study, this index was found to be reliable (*α* = 0.814).
- **Self‐Efficacy (8 Items)**: This scale measures an individual's tendency to perceive their own ability to execute various tasks. The scale is validated based on the research by Chen et al. (2001). It is measured on a Likert scale ranging from 1 (Not at all) to 5 (Very much). In the present study, this scale demonstrated high reliability (*α* = 0.937).
- **Conformity Tendency (11 Items)**: This scale examines an individual's tendency to adopt the attitudes, behaviors, or norms of the group to which they belong, even when these do not align with their personal values or beliefs, in an effort to conform to the social environment. The scale is validated based on the research by Mehrabian and Stefl (1995). It is measured on a Likert scale ranging from 1 (Not true at all for me) to 7 (Very true for me). Some items are reverse‐coded. In the current study, it was found that the second statement (“I would be the last person to change my opinion during a heated discussion on a controversial issue”) did not behave consistently with the rest of the items. Therefore, the final score was calculated as the average of the remaining ten items, following reverse coding of Items 25, 27, and 29. This scale demonstrated acceptable reliability (*α* = 0.783).
- **Emergency Preparedness Index (PI) (14 Items)**: This scale is calculated as the sum of items in which the respondent indicates having performed one or more of 14 recommended emergency preparedness actions. The scale is validated based on prior research (Bodas et al. 2015). In the present study, the scale demonstrated acceptable reliability (*α* = 0.773).
- **Sociodemographic Variables**: Gender (categorical‐nominal), age (continuous), income (categorical‐ordinal), education (categorical‐ordinal), religiosity (categorical‐ordinal), political orientation (categorical‐ordinal), country of birth (categorical‐nominal), relationship status (categorical‐dichotomous), number of children (categorical‐dichotomous and continuous), and place of residence (categorical‐nominal).

In line with prior literature, we examined gender and trust as predictors of behavioral intentions. Our approach acknowledges that skepticism toward authorities may reflect not only susceptibility to misinformation but also rational critical engagement, particularly considering prior crises where official messages were inconsistent. Gendered differences in preparedness and risk‐taking are interpreted here as patterns influenced by social roles and decision‐making strategies under uncertainty, rather than essentialist traits.

### 2.4 Tool Validation

The first phase of the study employed the e‐Delphi method to conduct an online expert panel of Israeli professionals, academics, and practitioners with over 10 years of experience in fields such as emergency and disaster management, risk communication, public information, and resilience. The panel aimed to reach consensus (defined as ≥ 70% agreement) on relevant emergency scenarios, key counterproductive public behaviors that could hinder local authority operations, and effective public information messages to mitigate such behaviors. Through three iterative rounds, the panel developed widely agreed‐upon inputs that informed the design of the subsequent public survey. Based on the panel's findings and a literature review, the research team finalized the questionnaire and intervention materials.

In addition, a preliminary pilot study involving 30 participants from the target population was employed to verify the internal validity of indices in the final tool. Cronbach's alpha values were computed to estimate the level of consistency in participants’ response to items belonging to the same construct. Threshold levels of consistency of 0.700 were achieved for all constructs.

### 2.5 Data Analysis

The statistical analysis of the research findings was conducted using SPSS software (version 29). The analysis included both descriptive statistics and inferential statistics to test the research questions. Prior to conducting the statistical tests, the various indices were constructed by assessing internal consistency reliability (Cronbach's alpha). The statistical analysis for this study was conducted in several stages to explore relationships between variables and identify underlying behavioral patterns. First, factor analysis was employed to determine how individual behavioral elements clustered together, allowing the construction of composite behavioral indices. To examine associations between variables, we applied a combination of ANOVA, independent samples *t‐*tests, and Spearman correlation analyses, depending on the level of measurement and distribution characteristics. To further explore predictors of behavior, multivariate linear regression models were conducted for each of the three behavioral factors identified through factor analysis. Prior to modeling, multicollinearity was tested and ruled out, and assumptions of homoscedasticity were assessed to ensure the appropriateness of the models. Statistical significance was set at *p* < 0.05 for all analyses, unless adjustments for multiple comparisons were warranted.

### 2.6 Ethical Considerations

The study was approved by the Ethical Committee of Tel Aviv University (approval No. 000846‐1‐2, dated May 22, 2024). Data collection was conducted following informed consent and under full anonymity.

## 3 Results

### 3.1 Descriptive Findings

The final sample included 1509 participants, approximately 50% of whom were women. The mean age of participants was 44 years (SD = 16). The distribution of sociodemographic variables is presented in Table 1.

**TABLE 1:** Distribution of sociodemographic variables in the sample.

Variable	*N* (%)	Variable	*N* (%)
**Gender**		**Affiliation with religion**	
Female	759 (50.3)	Secular	699 (46.3)
Male	750 (49.7)	Traditional	481 (31.9)
**Age (M = 43.96, SD = 15.97)**		Religious	194 (12.9)
18–30	380 (25.2)	Very religious	135 (8.9)
31–50	609 (40.4)	**Income**	
51 or above	519 (34.4)	Less than average	595 (39.4)
Missing	1 (< 0.1)	Average	390 (25.8)
**Education**		More than average	495 (32.8)
Up to K–12	40 (2.7)	Missing	29 (1.9)
K–12 (Matriculation)	412 (27.3)	**Birthplace**	
Vocational	344 (22.8)	Israel	1314 (87.1)
Academic	713 (47.2)	Other	195 (12.9)
**Familial status**		↳Immigrated before 1991	116 (7.7)
Coupled	1022 (67.7)	↳Immigrated after 1991	78 (5.2)
Not coupled	487 (32.3)	**Place of residence**	
**Children**		Greater Jerusalem	189 (12.5)
No	717 (47.5)	Greater Tel Aviv	556 (36.8)
Yes	792 (52.5)	Haifa and North	326 (21.6)
**No. of children**		South and Coastal Plains	276 (18.3)
0	717 (47.5)	HaSharon region	162 (10.7)
1–2	567 (37.6)	**Political affiliation**	
3 or more	225 (14.9)	Left	51 (3.4)
		Center‐left	123 (8.2)
		Center	343 (22.7)
		Center‐right	409 (27.1)
		Right	571 (37.8)

In the general sample, regarding perceived threat indices, approximately one‐third (*n* = 517, 34.3%) of participants assessed the likelihood of the scenario to which they were exposed as moderate. The scenarios perceived as most likely were civil disturbances (M = 3.21, SD = 1.04) and wildfires (M = 3.15, SD = 0.92), while the scenario perceived as least likely was flooding (M = 2.22, SD = 0.93), according to an ANOVA test (F = 56.48, df = 4, *p* < 0.001).

In the general sample, the perceived intrusiveness of the various threats was low, with approximately 68% indicating low or very low intrusiveness. The scenario generating the highest perceived intrusiveness was a hazardous materials (HazMat) event (M = 2.50, SD = 0.99), and the lowest was a wildfire scenario (M = 1.86, SD = 0.77) (F = 27.76, df = 4, *p* < 0.001).

The perceived severity of the scenarios in the general sample tended to be moderate or even low, with the question referring to the severity of the scenario for the respondent personally. Analysis of the severity index (which averages the two severity‐related items) indicates that the most severe scenario was a HazMat event (M = 2.87, SD = 0.82), while the least severe was extreme weather (M = 2.08, SD = 0.69) (F = 60.18, df = 4, *p* < 0.001).

In the general sample, most respondents were not particularly concerned about the listed emergency situations, with approximately 57% reporting low or very low levels of concern. The scenario evoking the greatest concern was civil disturbances (M = 2.97, SD = 1.14), followed by wildfires (M = 2.50, SD = 1.17) and HazMat events (M = 2.51, SD = 1.23) (F = 37.55, df = 4, *p* < 0.001).

Participants' level of trust in emergency guidelines and the response capacity of rescue forces was moderate to high, with ∼46% indicating “high” or “very high” levels of trust in the emergency instructions and ∼35% in the first responders capacity to respond. Participants expressed the highest levels of trust in the guidelines and emergency forces in the context of a wildfire scenario (M = 3.43, SD = 0.97 and M = 3.29, SD = 1.01, respectively), and the lowest levels in the context of civil disturbances (M = 3.05, SD = 1.05 and M = 2.86, SD = 1.02, respectively) (F = 8.52, df = 4, *p* < 0.001 and F = 7.28, df = 4, *p* < 0.001, respectively).

While participants in the different arms of the study did not differ significantly in their sense of self‐efficacy (F = 0.70, df = 4, *p* = 0.592), differences were found in their reported sense of preparedness across scenarios. The highest reported sense of preparedness was for extreme weather (M = 3.17, SD = 0.91), and the lowest for HazMat events (M = 2.43, SD = 0.94) and flooding (M = 2.62, SD = 0.95) (F = 28.99, df = 4, *p* < 0.001).

No significant differences were found between participants in the various study arms in terms of the conformity index (*p* = 0.097).

### 3.2 Main Dependent Variable—Behavioral Intention

Participants were asked to report their behavioral intention on several aspects of adherence to emergency instructions, seeking more information, acquiring emergency provisions, and engaging in unwanted behavior. Table 2 summarizes the frequency of each behavioral intent according to the studied scenarios.

**TABLE 2:** Distribution (%) of participants responding they were “much” or “very much” likely to engage in behavioral intentions, according to scenarios.

Behavioral intent	General sample	Forest fires	Flooding	HazMat	Extreme weather	Social disturbance
Seek additional information from emergency services	72.5	74.1	70.9	80.8	70.5	66.6
Acquire provisions for emergencies	69.6	65.8	59.9	74.2	81.5	66.2
Reach out to the local authority for more information	63.9	63.5	62.2	68.8	65.2	59.6
Resort to self‐help, not wait for to the rescue service	63.9	73.4	63.6	61.3	54.8	46.4
Leave the house, despite the emergency, if I felt danger to myself and my family from staying indoors	51.2	67.4	54.6	41.0	53.3	40.4
Attempt to get equipment and provisions at any cost, even if they are missing in the stores	39.5	35.2	34.4	45.3	49.3	33.1
Await further instruction from the local authority before taking any action, even if the instruction would be delayed for several hours	25.5	14.0	25.2	25.8	28.8	33.8
Spread updated, but unfounded information pertaining to the emergency	10.3	11.0	8.6	11.6	9.6	10.3
Attempt to reach the scene of the event, despite instruction	3.6	5.3	4.3	2.6	2.9	2.4

The descriptive distribution of the participants' behavioral intention was examined, both in the general sample and across the different scenarios. Based on this analysis and a factor analysis, it was decided to generate three distinct factors of behavioral intention. The first is **Self‐help**, which includes three behavioral items describing a preference for self‐help over reliance on external assistance (Cronbach's alpha = 0.605): “Taking action to help oneself rather than waiting for emergency services,” “Waiting for instructions from the local authority before taking any action, even if these are delayed by several hours” (reverse coded), and “Leaving the house despite the emergency, if feeling that staying at home poses a risk to oneself and one's family.” The second is **Knowledge‐seeking and preparedness behavior**, which includes four behavioral items describing the intention to seek additional information and to equip oneself for coping with the emergency (*α* = 0.686): “Seeking additional information from the local authority,” “Seeking additional information from emergency organizations,” “Equipping oneself with emergency supplies,” and “Attempting to acquire recommended equipment and resources at all costs, even if they are scarce in stores or retail chains.” The third factor was named **Counterproductive behavior**, and it includes two behavioral items describing actions that could endanger the participant and create additional complications for those managing the event (*α* = 0.449): “Disseminating current but unverified information related to the emergency” and “Arriving at the scene of the event, contrary to official instructions.” Despite the low alpha value of the two items, it was decided to construct the index due to the existence of sufficiently good correlations between them (R = 0.33, *p* < 0.001).

In the general sample (*N* = 1509), respondents who were exposed to rumors but not to explanatory information and guidelines reported an intention to engage in self‐help behavior at a level similar (M = 3.64, SD = 0.76) to that of respondents who were also exposed to explanatory information and guidelines (M = 3.66, SD = 0.74), according to an independent samples *t*‐test (*t* = 0.41, df = 1507, *p* = 0.682). Respondents who were exposed to rumors but not to explanatory information and guidelines reported a slightly higher intention to engage in knowledge‐seeking and preparedness behavior (M = 3.47, SD = 0.83) compared to respondents who were also exposed to explanatory information and guidelines (M = 3.40, SD = 0.89); however, this difference was not statistically significant (*t* = −1.68, df = 1507, *p* = 0.093). Respondents who were exposed to rumors but not to explanatory information and guidelines reported a higher intention to engage in counterproductive behavior (M = 1.72, SD = 0.75) compared to respondents who were also exposed to explanatory information and guidelines (M = 1.62, SD = 0.76), and this difference was statistically significant (*t* = −2.625, df = 1507, *p* = 0.009).

The data analysis further indicates that there are differences between the various scenarios in relation to the different behaviors. The scenario that elicits the highest knowledge‐seeking and preparedness behavior is the HazMat scenario, followed by extreme weather, wildfires, urban flooding, and finally, civil unrest. The differences between the scenarios are statistically significant, according to an ANOVA test (F(4) = 11.57, *p* < 0.001). The scenario that generates the highest behavioral intention for self‐help is the wildfire scenario, followed by extreme weather, urban flooding, HazMat, and civil unrest. The difference is statistically significant (F(4) = 27.13, *p* < 0.001). The scenario that most encourages counterproductive behavioral intention is wildfires, followed by urban flooding, extreme weather, HazMat, and civil unrest. The difference is statistically significant (F(4) = 3.77, *p* = 0.005).

#### 3.2.1 Factor 1—Self‐Help

This factor was found to be negatively correlated with threat intrusiveness (R = −0.10, *p* < 0.001), the worry index (R = −0.09, *p* < 0.001), the emergency trust index (R = −0.07, *p* = 0.008), and the conformity index (R = −0.23, *p* < 0.001). It was positively correlated with the sense of preparedness index (R = 0.11, *p* < 0.001) and the self‐efficacy index (R = 0.19, *p* < 0.001).

Men demonstrated a higher behavioral intention for self‐help (M = 3.52, SD = 0.84) than women (M = 3.42, SD = 0.84), according to an independent samples *t*‐test (*t*(1507) = 3.88, *p* < 0.001). A marginally significant trend was observed among participants who are parents, who demonstrated higher behavioral intention for self‐help (M = 3.47, SD = 0.86) compared to those who are not in a relationship (M = 3.39, SD = 0.86), based on an independent samples *t*‐test (*t*(1507) = 1.88, *p* = 0.060).

At the threshold of significance (not significant after correction for multiple comparisons), participants with a center‐right political affiliation showed slightly higher behavioral intention for self‐help (M = 3.45, SD = 0.86) than those with a center‐left affiliation (M = 3.31, SD = 0.84), as indicated by an independent samples *t*‐test (*t*(1495) = 2.05, *p* = 0.041). Similarly, participants who are not secular (i.e., traditional, religious, or very religious) demonstrated slightly higher behavioral intention for self‐help (M = 3.48, SD = 0.86) compared to center‐left affiliated participants (M = 3.38, SD = 0.86), according to an independent samples *t*‐test (*t*(1507) = −2.42, *p* = 0.016).

#### 3.2.2 Factor 2—Knowledge‐Seeking and Preparedness Behavior

This factor was found to be positively correlated with threat intrusiveness (R = 0.10, *p* < 0.001), perceived severity (R = 0.15, *p* < 0.001), trust in guidance (R = 0.15, *p* < 0.001), concern index (R = 0.14, *p* < 0.001), trust in emergency organizations (R = 0.14, *p* < 0.001), self‐efficacy index (R = 0.20, *p* < 0.001), emergency preparedness index (R = 0.23, *p* < 0.001), and trust in the government during the “Iron Swords” war (R = 0.07, *p* = 0.005).

Women demonstrated a higher behavioral intention to seek knowledge and acquire emergency supplies (M = 3.76, SD = 0.69) than men (M = 3.54, SD = 0.78), according to an independent samples *t*‐test (*t* = −5.93, df = 1480.86, *p* < 0.001). At the threshold of statistical significance (not significant after correction for multiple comparisons), participants in a relationship exhibited a higher behavioral intention to seek knowledge and acquire emergency supplies (M = 3.68, SD = 0.73) than those not in a relationship (M = 3.59, SD = 0.78), according to an independent samples *t*‐test (*t* = −2.01, df = 897.74, *p* = 0.045). Participants who are parents demonstrated a significantly higher behavioral intention to seek knowledge and acquire emergency supplies (M = 3.73, SD = 0.69) than participants who are not in a relationship (M = 3.56, SD = 0.79), as shown by an independent samples *t*‐test (*t* = −4.34, df = 1433.02, *p* < 0.001).

Participants identifying with a center‐right political orientation showed a higher behavioral intention to seek knowledge and acquire emergency supplies (M = 3.68, SD = 0.75) compared to those identifying with a center‐left orientation (M = 3.43, SD = 0.74), according to an independent samples *t*‐test (*t* = −4.13, df = 1495, *p* < 0.001). A trend toward statistical significance was observed among non‐secular participants (traditional, religious, very religious), who demonstrated higher behavioral intention to seek knowledge and acquire emergency supplies (M = 3.68, SD = 0.75) compared to secular participants (M = 3.61, SD = 0.75), as shown by an independent samples *t*‐test (*t* = −1.85, df = 1507, *p* = 0.064).

#### 3.2.3 Factor 3—Counterproductive Behavior

This factor was found to be negatively correlated with the self‐efficacy measure (R = −0.08, *p* = 0.002) and age (R = −0.11, *p* < 0.001), and positively correlated with the measure of conformism (R = 0.19, *p* < 0.001) and trust in the government during the “Iron Swords” war (R = 0.10, *p* < 0.001).

Men demonstrated a higher level of behavioral intention toward counterproductive behavior (M = 1.75, SD = 0.80) compared to women (M = 1.59, SD = 0.70), according to an independent samples *t*‐test (*t* = 4.19, df = 1475.99, *p* < 0.001). Participants who were not secular (i.e., traditional, religious, or very religious) exhibited a higher level of behavioral intention toward counterproductive behavior (M = 1.72, SD = 0.79) compared to secular participants (M = 1.62, SD = 0.72), according to an independent samples *t*‐test (*t* = −2.65, df = 1501.81, *p* = 0.008).

Participants with a center‐right political orientation demonstrated a higher level of behavioral intention toward counterproductive behavior (M = 1.70, SD = 0.77) compared to those with a center‐left orientation (M = 1.51, SD = 0.68), according to an independent samples *t*‐test (*t* = −3.43, df = 235.28, *p* < 0.001).

### 3.3 Multivariate Analysis

Three multiple linear regression analyses were conducted for each of the behavioral factors. In each analysis, variables that were found to be associated with the dependent variable in the univariate analysis were included, following the negation of multicollinearity and assessment of homoscedasticity. In all analyses, gender, age, exposure to guidelines, and scenario types (as dummy variables) were included for the purpose of standardization. The full results are presented in Appendix 2 of the Supporting Information.

#### 3.3.1 Factor 1—Self‐Help

The model was found to be significant (F = 19.79, *p* < 0.001), explaining 18.5% of the variance in the variable. The factors that remained significant predictors of the intention to engage in self‐help behavior were gender (*β* = −0.088), trust in first responders (*β* = −0.158), perceived preparedness (*β* = 0.086), self‐efficacy (*β* = 0.088), conformism (*β* = −0.207), exposure to a wildfire scenario (*β* = 0.151), and exposure to a civil disorder scenario (*β* = −0.188), with political affiliation also contributing to a lesser extent.

#### 3.3.2 Factor 2—Knowledge‐Seeking and Preparedness Behavior

The model was found to be significant (F = 17.84, *p* < 0.001), explaining 19.5% of the variance in the variable. The factors that remained significant predictors of the intention to engage in knowledge‐seeking and preparedness behavior were gender (*β* = 0.128), threat intrusiveness (*β* = 0.060), perceived severity (*β* = 0.081), concern (*β* = 0.114), trust in first responders (*β* = 0.082), self‐efficacy (*β* = 0.131), being a parent to children (*β* = 0.074), political affiliation (*β* = 0.064), and the emergency preparedness index (*β* = 0.205). In addition, exposure to wildfire, urban flooding, and civil disorder scenarios was found to be significant predictors.

#### 3.3.3 Factor 3—Counterproductive Behavior

The model was found to be significant (F = 9.91, *p* < 0.001), explaining 7.4% of the variance in the variable. The factors that remained significant predictors of the intention to engage in counterproductive behavior were gender (*β* = −0.107), age (*β* = −0.086), conformism (*β* = 0.144), political affiliation (*β* = 0.099), and exposure to official guidelines (*β* = 0.074). Only the wildfire scenario remained a significant predictor.

## 4 Discussion

The study reveals a complex picture of public behavior in Israel during emergencies, marked by a significant gap between threat perception and compliance with official guidelines—especially those from local authorities, compared to national emergency agencies. While the public demonstrates fairly reasonable threat awareness, intentions to follow instructions, particularly those involving seeking help or information from local authorities, remain limited. Nonetheless, public information campaigns are vital in mitigating impulsive or hazardous behavior, even if they do not always encourage proactive civic actions. These results raise key questions about the efficacy of municipal communication, public trust, and areas for improvement in both local and national risk communication strategies. The Israeli public differentiates between types of emergencies, perceiving HazMat and fires as more dangerous than floods or extreme weather, aligning with literature on risk perception, which varies based on threat frequency and sociocultural factors (Bodas et al. 2019; Bodas 2021; Lefkowitz and Bodas 2023; Bodas et al. 2022; Jiao et al. 2023; Schoessow et al. 2022).

Despite high threat perception, only 64% of respondents indicated they would seek guidance from local authorities, compared to 72.5% who would contact national emergency bodies. This points to trust and communication challenges. Local authorities, despite their proximity to communities, may struggle with logistical and technological constraints that hinder real‐time communication, especially in a context of unitary government, such as the one existing in Israel. In addition, social media amplifies conflicting messages, often overshadowing local messaging and creating dissonance when information from local, national, and informal sources diverges (Fathollahzadeh et al. 2024; Boholm 2019; Lerøy Sataøen and Eriksson 2023; Cole and Fellows 2008).

Effective emergency communication depends not only on message content but also on the credibility of the source, timing, and delivery method. Inconsistent or unclear messages reduce trust, potentially triggering unwanted behavior or disregard for safety instructions. Our regression models indicated that conformity was positively associated with rumor‐sharing. This suggests that reliance on trusted community figures may help redirect conformity dynamics toward compliance with safety instructions. Municipal strategies could therefore test whether integrating local opinion leaders as message amplifiers reduces rumor circulation and increases alignment with official guidance. In addition, behavioral differences by age and gender in our sample support the need for differentiated communication strategies. For example, women were more likely to report preparedness actions, while men reported higher self‐help and counterproductive behaviors. Future field studies could evaluate whether tailoring channels (e.g., broadcast media for older adults, social media for younger groups) increases uptake of recommended protective actions.

Local authorities must also operate under uncertainty, which may lead to the dissemination of incomplete or inaccurate information. Combined with poor coordination among emergency bodies, this weakens message coherence. Our results showed that trust in national authorities was consistently higher than in local governments, suggesting that discrepancies between municipal and national messaging may undermine compliance. This implies that testing joint message protocols in coordinated drills could help reduce dissonance and strengthen credibility across levels of authority. These findings point to a need for further research into how trust‐building initiatives during non‐crisis periods influence behavioral responses during emergencies. Longitudinal or field‐based studies could clarify whether proactive municipal engagement enhances credibility and reduces counterproductive behaviors when crises occur.

The study shows that while information campaigns have limited influence on proactive behaviors like preparedness or information seeking, they are significantly effective in reducing counterproductive behaviors, such as rumor‐spreading or approaching danger zones. This confirms that public messaging acts more as a harm‐reduction tool than a proactive motivator, consistent with prior studies on emergencies involving unconventional terrorism and the “worried well” (Bodas et al. 2021).

Public responses also vary by scenario. “Natural‐technological” events such as HazMat or severe weather elicit active information‐seeking and preparedness, while human‐induced events (e.g., civil unrest or violence) often result in passivity or avoidance. This may relate to perceived control, political framing, and trust in institutions. Natural threats are seen as manageable through preparation, whereas human threats are viewed as chaotic or emotionally charged, reducing public initiative and increasing ambiguity (Shapouri et al. 2023).

Hence, communication must be tailored to threat type. For predictable threats, guidance should focus on practical steps; for unpredictable or politicized threats, messages should aim to restore control and clarify leadership. A “one‐size‐fits‐all” approach may provoke resistance or anxiety. Scenario‐specific messaging may improve compliance and reduce negative reactions.

Sociodemographic characteristics also shape behavior. Women are more likely to seek information and prepare emergency kits, while men show higher self‐confidence and risk‐taking. These findings are consistent with the literature indicating that women emphasize household preparedness (Wester et al. 2024; Vasseur et al. 2015), while men prioritize operational readiness (Mulilis 1999; Cvetković et al. 2018). In recent unpublished data, Israeli men rated themselves as more technically prepared (e.g., knowing where water and gas valves are), while women engaged more in early‐stage preparations such as preparing the household for emergencies. These gendered patterns have important implications for risk communication strategy. Women's higher rates of preparedness behaviors and information‐seeking, combined with their typical roles as family coordinators, suggest they may serve as effective conduits for emergency information within households and communities. Conversely, men's tendency toward overconfidence and counterproductive behaviors indicates a need for messaging that explicitly addresses risk perception biases without triggering defensive reactions. Targeting communication through channels and community figures that resonate with different gender roles—while being mindful not to reinforce harmful stereotypes—may enhance overall community preparedness. For example, engaging women in low‐income communities in the Israeli periphery might increase compliance patterns by effectively transferring responsibility over emergency response from men (who tend to be overconfident about their technical prowess) to women (who tend to comply better partly due to their positioning as family anchors). This approach could help address some of the other biases associated with socioeconomic background or ethnicity that we observed in our data.

Other key variables—such as marital status, religiosity, and political affiliation—also influenced responses. Individuals with strong political or ideological identities sometimes responded with either elevated trust or skepticism, depending on the threat and the message. This points to the importance of designing communication that resonates with identity markers like gender, age, language, and political worldview.

Conformism also emerged as a significant factor. It was negatively associated with self‐help behaviors and positively with rumor‐sharing, suggesting that in uncertain times, people often follow the crowd—even against expert advice. This reinforces the importance of clear, credible leadership to provide a behavioral anchor during emergencies (Liao et al. 2018).

This study demonstrates that municipal informational messages can significantly reduce counterproductive behaviors but are less effective in promoting proactive preparedness. These results extend established theoretical models by showing that harm‐reduction, rather than motivation, is the primary function of local‐level messaging. The regression findings, for example, indicate that conformity predicts rumor‐sharing, suggesting that authorities should consider enlisting trusted community opinion leaders to anchor behavior during uncertainty. Because these recommendations emerge from correlational patterns, they should be framed as hypotheses for future testing rather than prescriptive conclusions.

### 4.1 Case Study: The Jerusalem Forest Fires, April 2025

In April 2025, during the final phase of this study, a major wildfire broke out in the Jerusalem Hills—one of the most severe fires in Israel's history. Sparked by extreme weather, including high winds and dry conditions, the blaze spread rapidly across 7000–10,000 dunams of forest. Over 100 firefighting teams and six aircraft worked for more than 20 h to contain the fire. An emergency protocol was activated, and international assistance was requested from Greece, Italy, and Cyprus. Several communities were evacuated. While there were no fatalities, three firefighters and one police officer suffered smoke inhalation. The most significant damage was ecological, with widespread destruction of natural habitat.

The incident closely resembled the wildfire scenario examined in this study, providing a real‐world validation of the findings. As predicted, residents showed high compliance with evacuation orders and refrained from entering danger zones. Some even volunteered to assist response efforts, demonstrating a strong sense of self‐efficacy and civic responsibility.

Clear and timely communication from both local authorities and national agencies played a critical role. Alerts and instructions were issued across multiple platforms—SMS, social media, and broadcast media—focusing on actionable steps like evacuations and shelter locations. Consistent and credible messaging helped avoid confusion and discouraged misinformation.

The event also highlighted the importance of public trust. Effective coordination among emergency services and municipalities fostered confidence in institutional response, encouraging compliance. Communities with prior experience or preparedness training responded more rapidly and decisively. In contrast, unfamiliar or less‐engaged communities exhibited more hesitation.

In sum, the Jerusalem Hills wildfire served as a powerful case study supporting this research's findings on public behavior, risk communication, and the role of local authorities. It underscored the value of scenario‐based planning, strong institutional messaging, and public trust in managing complex emergencies.

### 4.2 Theoretical Contribution

This study contributes to the broader risk communication literature by situating municipal‐level messaging within established theoretical frameworks of public response to emergencies. The Protective Action Decision Model (PADM) emphasizes how individuals move from environmental cues and warnings to protective behavioral decisions through the mediating roles of risk perception, protective action perception, and situational facilitators and impediments (Lindell and Perry 2012). Our findings support the PADM proposition that trust in authorities and perceived efficacy are central drivers of behavioral intentions, while also showing that municipal authorities are not perceived as equally credible sources compared to national institutions. This contextual nuance underscores the importance of tailoring PADM applications to local governance structures.

The results also resonate with the Social Amplification of Risk Framework (SARF), which posits that the flow of information through social, institutional, and cultural channels can amplify or attenuate public responses (Kasperson et al. 2022). We observed that municipal informational messages attenuated counterproductive behaviors such as rumor‐spreading and convergence on hazard sites, even in the presence of rumor exposure. This suggests that municipal actors can function as critical “amplification stations,” moderating the spread of misinformation and shaping the social interpretation of risk at the community level.

Finally, the patterns identified in this study align with dual‐process models of risk perception, which differentiate between affective, intuitive responses (“risk‐as‐feeling”) and more deliberative, cognitive assessments (“risk‐as‐analysis”) (Gray and Ropeik 2002). Scenario‐specific differences—for example, heightened compliance in response to wildfires versus lower initiative during civil unrest—illustrate how emotional salience and perceived controllability jointly shape behavior. Municipal messaging appears particularly effective in guiding affect‐driven responses toward safer outcomes, even when it does not fully motivate deliberative preparedness actions.

Taken together, these linkages extend established theories by highlighting the contextual role of municipal authorities in shaping behavioral intentions during emergencies. While prior research has largely focused on national‐level communication, our results demonstrate that trust dynamics, scenario type, and demographic predictors must also be considered at the local level to fully understand public responses to emergency communication. This municipal lens offers a novel contribution to both theory and practice in risk communication.

### 4.3 Limitations

This study has several limitations that should be considered when interpreting the findings. First, while the sample was designed to be representative of the adult Israeli population across key sociodemographic characteristics, recruitment through an online panel may have systematically excluded individuals with limited digital access or lower digital literacy. These groups are often among the most vulnerable during emergencies, and their perspectives are especially important for designing inclusive communication strategies.

Second, the reliance on hypothetical scenarios, although grounded in real‐world events, constrains ecological validity. Such vignettes cannot fully reproduce the emotional intensity, uncertainty, and time pressure that characterize actual crises, and this may have influenced participants' responses. Relatedly, our reliance on self‐reported behavioral intentions—commonly used proxies in risk communication research—may not directly translate into observed behaviors under real emergency conditions. Social desirability and self‐reporting biases may also have shaped some responses.

Finally, the cross‐sectional design precludes causal inference and does not capture dynamic processes such as how trust, message fatigue, or repeated exposure to crises may evolve over time. To address these limitations, future research should employ longitudinal designs, field experiments, or real‐time observational methods, and explicitly include populations with limited digital access. Mixed‐method approaches may also help capture the nuanced social and emotional dimensions of emergency response.

Despite these constraints, the present study provides valuable insight into the role of municipal messaging in shaping public behavior, and it highlights concrete directions for strengthening risk communication strategies at both the local and national levels.

## 5 Conclusions

The findings of this study suggest that while the Israeli public generally demonstrates sound awareness of emergency risks, there remains a notable gap between this awareness and the tendency to rely on local authorities for real‐time guidance. Informational messages appear to be more effective in curbing harmful behaviors than in encouraging proactive preparedness, pointing to a potential need for communication strategies that focus as much on harm prevention as on motivating action. The variability in public responses across different types of emergency scenarios—particularly between technological and human‐induced threats—raises questions about the appropriateness of uniform messaging strategies. In addition, sociodemographic factors such as age, gender, political orientation, and cultural background appear to influence behavioral intentions, indicating that more nuanced, audience‐specific communication approaches may be warranted.

The consistent emergence of gender as a significant predictor across behavioral factors deserves particular attention. Women demonstrated higher rates of knowledge‐seeking and preparedness behaviors, while men showed elevated levels of self‐help intentions and counterproductive behaviors such as rumor‐spreading. These patterns align with broader research on gendered responses to risk and suggest that municipal communication strategies might benefit from gender‐sensitive approaches. This does not mean reinforcing stereotypes, but rather recognizing existing patterns of household responsibility, information processing, and community engagement that differ across gender lines. For instance, since women are more likely to engage in preparedness behaviors and serve as family coordinators, they may be particularly effective targets for community preparedness programs and peer‐to‐peer communication initiatives. At the same time, messaging directed toward men might need to explicitly address overconfidence biases and emphasize the social value of protective behaviors rather than technical prowess. Future research should explore whether gender‐tailored communication strategies can improve overall community preparedness without reinforcing harmful gender norms.

In conclusion, municipal informational messaging plays a critical role in emergency management, functioning primarily as a tool to curb counterproductive behaviors such as rumor‐spreading and hazard convergence. Its effects on proactive preparedness are more limited, underscoring the need for complementary strategies that foster trust, build self‐efficacy, and engage communities before crises occur. Tailored, scenario‐specific messaging and proactive trust‐building during non‐emergency periods may enhance local credibility. Future research should evaluate these strategies in real‐world settings, using longitudinal and field designs to strengthen causal inference and ecological validity. By situating communication at the municipal level, this study contributes to a more nuanced understanding of public behavior in emergencies and provides practical pathways for improving community safety and resilience.

## Author Contributions

Conceptualization: Moran Bodas. Data curation: Moran Bodas and Omar Watad. Formal analysis: Moran Bodas. Funding acquisition: Moran Bodas and Stav Shapira. Investigation: Moran Bodas, Omar Watad, and Stav Shapira. Methodology: Moran Bodas, Omar Watad, and Stav Shapira; Supervision: Moran Bodas and Stav Shapira. Validation: Moran Bodas, Omar Watad, and Stav Shapira. Writing – original draft: Moran Bodas and Omar Watad. Writing – review and editing: Stav Shapira.

## Funding

This work was supported by the Bloomberg‐Sagol Center for City Leadership at Tel Aviv University.

## Conflicts of Interest

The authors declare no conflicts of interest.

## Supporting information




**Supporting Information**: risa70232‐supp‐0001‐SuppMat.docx

**Supporting Information**: risa70232‐supp‐0002‐SuppMat.docx

## Data Availability

Data are available upon reasonable request from the authors.
