# Supplementary material for: Municipal Risk Communication and Public Trust: Reducing Counterproductive Behaviors Across Emergency Scenarios
Source: Risk Anal. 2026 Mar 30;46(4):e70232. doi: 10.1111/risa.70232 (PMC13036389; doi:10.1111/risa.70232)
Supplement: Supplementary file 1 — Supporting Information: risa70232‐supp‐0001‐SuppMat.docx [file RISA-46-0-s002.docx]

**Appendix 1 – Intervention groups text**

Note: All Hebrew text in this appendix is provided with English translations in brackets for international readers.

**Selected Scenarios for the Study**
The following emergency scenarios were selected for the purposes of this study:

1. A large-scale forest fire
2. Urban flooding
3. Hazardous materials incident
4. Extreme weather event
5. Civil unrest

**Presentation Format of the Selected Scenarios**
For each scenario, a brief description was developed to present it to the study participants.

1. A large-scale forest fire

**
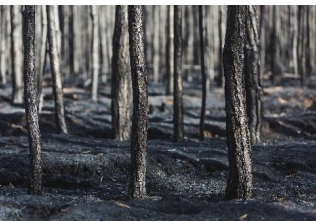
Below is a description of a hypothetical scenario involving a large forest fire near your place of residence. Please read it carefully and answer the questions that follow.**

Massive flames are consuming vast areas of forest adjacent to a populated area, spreading rapidly due to strong winds and extreme dryness. Firefighting teams are struggling to gain control over the blaze, but the volume of emergency calls and the shortage of resources are making the task increasingly difficult. Hundreds of families have been evacuated from their homes, and many report difficulty breathing due to the heavy smoke. Danny, a local resident, recounts: “Everything here is black. The smoke is suffocating us, and the
firefighters simply can’t stop it. I just hope our house makes it through the night.”

1. **Flooding in the city**

**
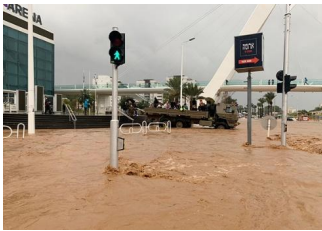
Below is a description of a hypothetical scenario involving the flooding of your city or place of residence. Please read it carefully and then respond to the subsequent questions.**

The heavy rains that have fallen in recent days have led to the flooding of large parts of the city, devastating its streets, claiming human lives, and leaving residents traumatized. Approximately 1,500 calls have been logged in the past 24 hours at the municipal emergency center from residents trapped in their homes, requiring rescue and assistance. As described by Rivka, a city resident:
"We’re stuck on the second floor of our house. There’s absolutely no way for us to get out. I don’t understand how anyone expects us to remain here like this."

1. **Hazardous Materials Incident**


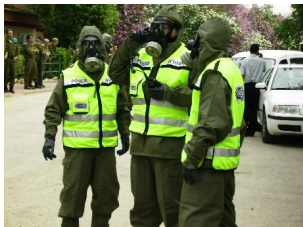
Below is a description of a hypothetical scenario involving the dispersion of hazardous material near your place of residence. Please read it carefully and respond to the questions afterward.

A massive leak of toxic chemical substances from an industrial plant causes widespread air contamination in the surrounding area. A dense cloud of dangerous gas spreads toward residential neighborhoods, and residents are instructed to remain indoors with windows closed. Local hospitals are overwhelmed with cases of respiratory distress, skin irritation, and poisoning. Anat, a mother of three, describes anxiously:
"My son started coughing non-stop, and his eyes are burning. We don’t know what to do, and the information is reaching us in dribs and drabs."

1. **Extreme Weather Event**


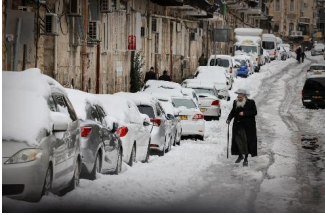
Below is a description of a hypothetical extreme weather event in your area. Please read it carefully and respond to the questions afterward.

An unusually severe winter storm strikes the region, bringing with it winds exceeding 120 km/h, heavy snowfall, and flooding. Thousands of households are cut off from electricity, and residents face extreme temperatures without heating. Roads are blocked due to fallen trees, and the accumulation of snow hinders emergency services from reaching isolated areas. Nir, an elderly man living alone, reports:
"I'm sitting at home wrapped in blankets, without heat, without light. I don’t know when this will end or if I’ll be able to survive it."


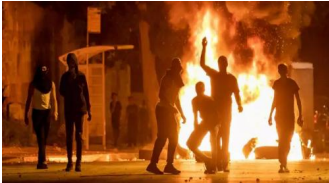
5**. Public Disturbances**Below is a description of a hypothetical scenario involving extreme weather conditions in your area. Please read it carefully and respond to the questions that follow.

Demonstrations that began in mixed cities across the country have escalated into violent riots, involving severe clashes between local groups and security forces. Roads have been blocked, stores looted, and vehicles set on fire. The unrest has spread to additional cities, with the police struggling to regain control of the situation. Many residents are afraid to leave their homes, and a pervasive sense of insecurity dominates the streets. Miriam, a resident of Jaffa, recounts:
"We hear explosions and shouting from the street. The children are scared, and we have no idea when this will end."

**Display of Rumors According to Scenario
For each scenario, a display of social media rumors was developed.**

[English translation in all rumor posts (only the image changes): *Don't be helpless and wait to be rescued. No one is coming. Take your fate at your own hands and do whatever it takes to protect your family. Police officials have already admitted that they cannot provide assistance and that things got out of control. What are you waiting for? For your name to be on the next memorial wall? In the picture: brave people who do not wait to be rescued*.]

1. **Large-scale forest fire**

**
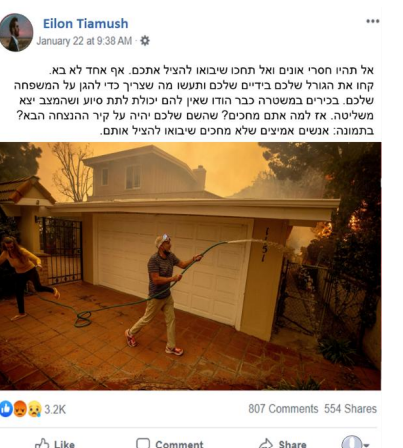
**

1. **
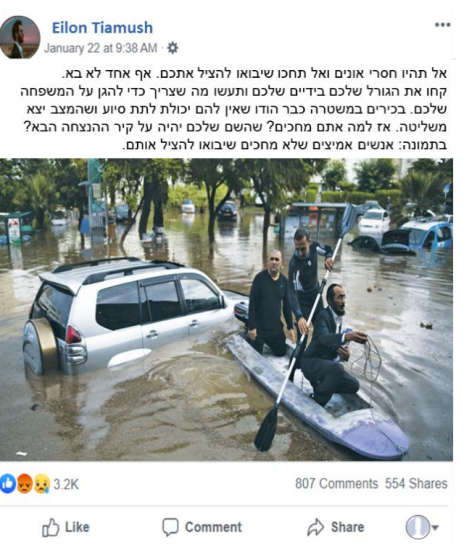
Flooding in the city**

1. **Hazardous Substances Event**


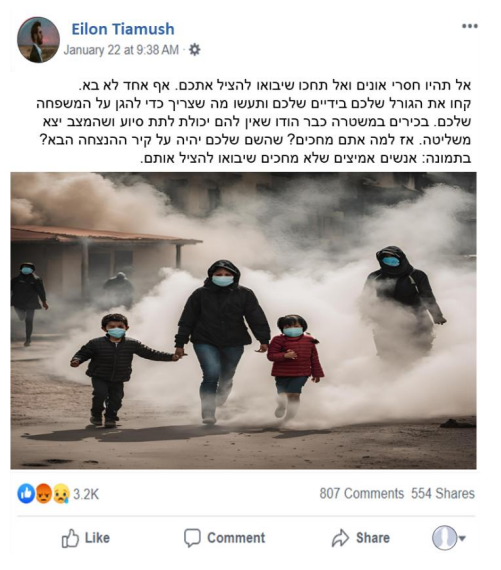


- 1. **Extreme Weather Event**


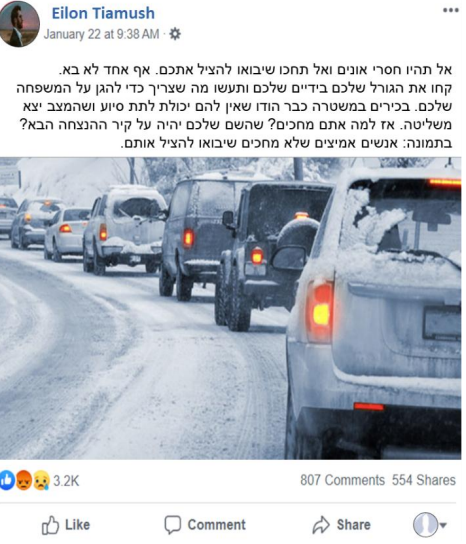


5**. Public Disturbances**
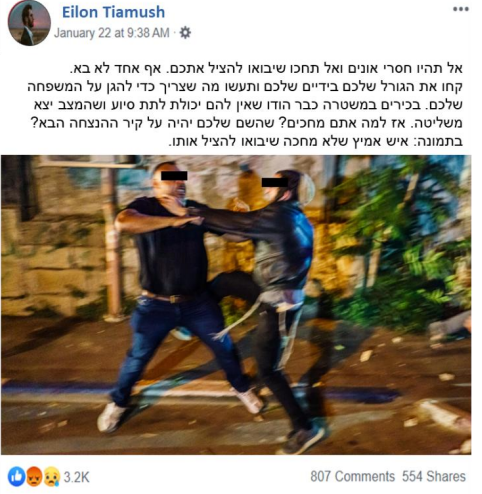


**Guidance and explanation presentation**
In all of the scenarios outlined above, a subset of participants was presented with behavioral guidelines. The phrasing of these guidelines takes into account principles of public communication and media relations aimed at reducing undesirable public behavior, as proposed by experts who partook in the eDelphi process preceding the study:

1. Utilizing messages tailored to different target audiences, for example, through professionals specializing in various fields (e.g., health services, emergency services, mental health) and community leaders or opinion influencers.
2. Encouraging volunteerism and enlisting the public in tasks coordinated by the local authority.
3. Explaining the consequences of inappropriate behavior and offering more constructive ways of coping with the situation.
4. Enhancing the public’s sense of efficacy, increasing their perceived control, and fostering a belief in their ability to cope with the situation.
5. Communicating messages with empathy.
6. Taking responsibility for the situation.
7. Setting realistic expectations with residents regarding what the authorities can and cannot do/achieve under current conditions.
8. Proactively providing information that may address a wide range of common questions.
   Avoid withholding information — if something is unknown, it should be clearly stated as such.

| 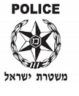  **The following are the official guidelines published for the public by the Israel Police and the local authority in light of the emergency situation:**  **Dear Residents,** The current emergency poses a real danger to you and your family. The local authority and the Israel Police, with the support of emergency and rescue services, are working to develop a clear situational awareness, to regain control, and to provide assistance to all those in need. We understand that you are facing considerable challenges during this difficult time. Nevertheless, you can contribute to the overall effort and ease the burden on emergency personnel — thereby enabling them to assist those most in need — by adhering to the following behaviors:   1. Follow only the instructions issued by official sources (Israel Police, the local authority, and recognized emergency and rescue organizations such as Magen David Adom) to ensure your safety and that of others. 2. Refrain from spreading unofficial or unverified information. 3. If you are located in a danger zone and cannot evacuate safely according to the instructions, take shelter in the nearest building and notify the municipal hotline or call 100. 4. The Israel Police, together with emergency services and the local authority, are working to reach all individuals in need of assistance. It is essential to comply with the instructions of personnel on the ground. Do not take unnecessary risks or attempt to handle dangerous situations on your own. 5. Individuals who wish to assist through volunteering in emergency-related tasks are requested to contact the municipal hotline.   Further instructions will be issued in due course. Please remain attentive to official communications via the media. |
| --- |
