# Supplementary material for: Municipal Risk Communication and Public Trust: Reducing Counterproductive Behaviors Across Emergency Scenarios
Source: Risk Anal. 2026 Mar 30;46(4):e70232. doi: 10.1111/risa.70232 (PMC13036389; doi:10.1111/risa.70232)

**Appendix 2 – Full results of multiple linear regression analyses**

**Factor 1 – Self-Help**


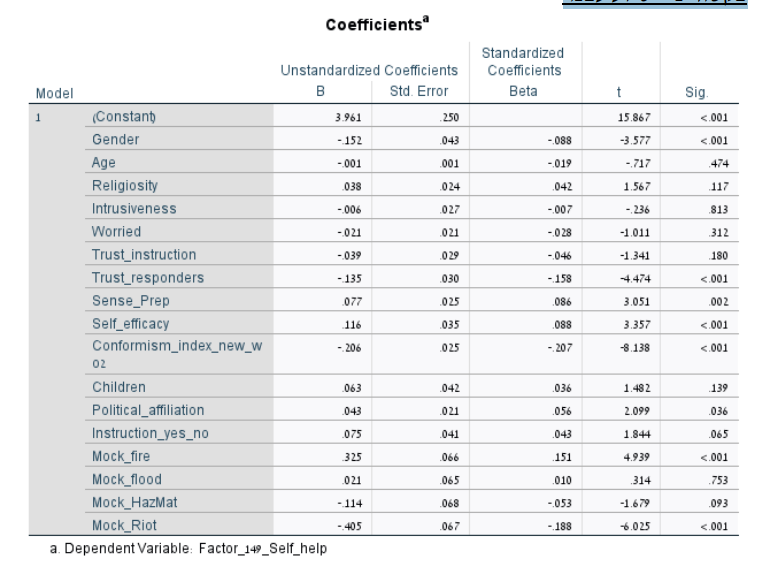


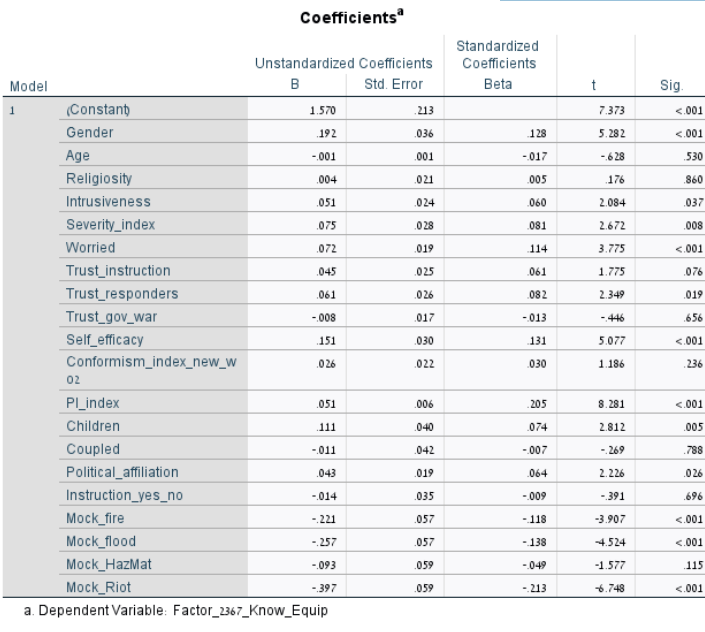
**Factor 2 – Knowledge-seeking and preparedness behavior**

**Factor 3 – Undesirable Behavior**


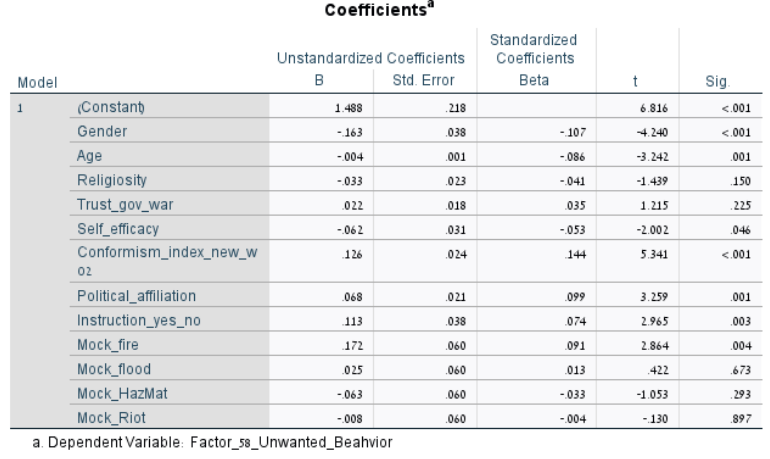

Supplement: Supplementary file 2 — Supporting Information: risa70232‐supp‐0002‐SuppMat.docx [file RISA-46-0-s001.docx]
